# Insight into the genetic diversity of *Anaplasma marginale* in cattle from ten provinces of China

**DOI:** 10.1186/s13071-017-2485-x

**Published:** 2017-11-13

**Authors:** Jifei Yang, Rong Han, Zhijie Liu, Qingli Niu, Guiquan Guan, Guangyuan Liu, Jianxun Luo, Hong Yin

**Affiliations:** 1 State Key Laboratory of Veterinary Etiological Biology, Key Laboratory of Veterinary Parasitology of Gansu Province, Lanzhou Veterinary Research Institute, Chinese Academy of Agricultural Sciences, Xujiaping 1, Lanzhou, Gansu 730046 People’s Republic of China; 2 Jiangsu Co-innovation Center for Prevention and Control of Important Animal Infectious Diseases and Zoonoses, Yangzhou, 225009 People’s Republic of China

**Keywords:** *Anaplasma marginale*, *msp4* gene, Msp1a tandem repeats, Genotypes, Cattle, China

## Abstract

**Background:**

*Anaplasma marginale* is an important tick-transmitted rickettsial pathogen of cattle, with worldwide distribution and an important economic impact. The genetic diversity of *A. marginale* strains has been extensively characterized in different geographical regions throughout the world, while information is limited on studies in China. This study was carried out to determine the prevalence and genetic diversity of *A. marginale* strains in cattle from ten provinces of China.

**Methods:**

A total of 557 blood samples from cattle were collected and screened for the occurrence of *A. marginale* by PCR based on the *msp4* gene. The partial *msp1a* gene containing tandem repeat sequences was further amplified from *msp4* positive samples. The Msp1a amino acid repeats were identified, and genetic variation of *A. marginale* strains was characterized based on the variation in the repeated portion of Msp1a.

**Results:**

Our results showed that 31.6% of 557 cattle were positive for *A. marginale*. The infection rates of *A. marginale* varied considerably from 0 to 96.9% in different sampling regions. Sequence analysis revealed that two *msp4* sequence variants of *A. marginale* exist in cattle. One hundred and three *msp1a* sequences were obtained and permitted to identify 42 Msp1a tandem repeats, 21 of which were not previously published for *A. marginale*. Moreover, 61 *A. marginale* genotypes were identified based on the structure of Msp1a tandem repeats.

**Conclusions:**

*Anaplasma marginale* is widely distributed in China and a high prevalence of infection was observed in cattle. The geographical strains of *A. marginale* were molecularly characterized based on the structure of Msp1a tandem repeats. Forty-two Msp1a tandem repeats and 61 genotypes of *A. marginale* were identified. This study, for the first time, revealed the genetic diversity of *A. marginale* strains in cattle in China.

## Background

*Anaplasma marginale* is an obligate intraerythrocytic pathogen that cause bovine anaplasmosis throughout the world [1]. It was first described in cattle by Sir Arnold Theiler in 1910, and is widely distributed in Africa, Asia, Australia, South and Central America, southern Europe, and the USA [2–5]. Animals infected by *A. marginale* develop a mild to severe life-threatening hemolytic disease, causing considerable economic loss to the cattle industry worldwide [5]. The organism can be transmitted biologically by ticks and mechanically by blood-sucking arthropods or blood-contaminated fomites [6]. Approximately 20 tick species, mainly of the genera *Rhipicephalus* and *Dermacentor*, have been recorded as vectors of *A. marginale* [7]. *Anaplasma marginale* is host-specific, and cattle and water buffaloes are highly susceptible to infection [6, 8, 9]. The animals that recover from acute anaplasmosis develop persistent infection and act as reservoirs for this causative agent [1].

To date, a great number of geographical strains of *A. marginale* have been identified on a global scale, which vary in genotype, virulence, antigenic characteristics and infectivity for ticks [6]. Characterization of the genetic diversity of *A. marginale* strains has been performed based on the variability of tandem repeat amino acid sequences located in the N-terminal region of the major surface protein (Msp) 1a, and numerous geographical Msp1a tandem repeats and genotypes were identified [10]. In China, *A. marginale* has been recognized for over 30 years, and *Rhipicephalus microplus* is considered to be the most important tick vector with a nationwide distribution [11, 12]. Despite the importance of bovine anaplasmosis, limited information is available for *A. marginale* in China. Previously, the occurrence of *A. marginale* was reported in several provinces, and only one Msp1a tandem repeat (GenBank: DQ811774) was identified in *A. marginale* strain HB-A8 from cattle [11–15]. The objective of this study was to determine the prevalence and genetic diversity of *A. marginale* strains in cattle from different geographical areas of China.

## Methods

### Study areas, sample collection and DNA isolation

This study was conducted between 2011 and 2015 in rural areas of 22 counties from ten provinces of China, including Inner Mongolia and Liaoning (north-east China); Hunan, Guangdong, Guangxi and Hainan (south-central China); Chongqing, Sichuan, Guizhou and Yunnan (south-west China). The sample sites are listed in Table 1. Animals for this study were randomly selected in two to three herds for each county. A total of 557 jugular blood samples were collected in vacutainer EDTA tubes from adult cattle. Genomic DNA was prepared from 300 μl blood samples using the Gentra Puregene Blood Kit (Qiagen, Beijing, China) following the protocols recommended by the producer. DNA was resuspended in the elution buffer provided in the commercial kit and stored at -20 °C until use.

**Table 1:** Detection of *A. marginale* in cattle from China, 2011–2015

	Location		No. tested	No. positive (%)
Area	Province	County		
South Central	Hunan	Yongzhou	18	2 (11.1)
		Linli	36	0 (0)
		Lianyuan	31	14 (45.2)
	Guangdong	Qingyuan	25	23 (92.0)
		Zhaoqing	24	19 (79.1)
		Maoming	50	1 (2.0)
	Guangxi	Baise	32	14 (43.8)
		Tianyang	26	22 (84.6)
		Chongzuo	12	7 (58.3)
	Hainan	Chengmai	46	0 (0)
	Subtotal		300	102 (34.0)
Southwest	Chongqing	Jiangjin	30	1 (3.3)
		Wanzhou	25	3 (12.0)
	Sichuan	Panzhihua	32	31 (96.9)
	Guizhou	Dushan	30	18 (60.0)
		Rongjiang	12	10 (83.3)
	Yunnan	Yanshan	29	1 (3.4)
		Ruili	17	0 (0)
		Fuyuan	8	3 (37.5)
	Subtotal		183	67 (36.6)
Northeast	Liaoning	Benxi	16	2 (12.5)
		Anshan	29	0 (0)
	Inner Mongolia	Xinbaerhuzuoqi	15	5 (33.3)
		Eerguna	14	0 (0)
	Subtotal		74	7 (9.5)
	Total		557	176 (31.6)

### PCR reactions

The extracted DNA was used for the amplification of *msp4* gene of *A. marginale* by nested PCR [16, 17]. Briefly, the primers MSP43 (5′-GGG AGC TCC TAT GAA TTA CAG AGA ATT GTT TAC-3′) and MSP45 (5′-CCG GAT CCT TAG CTG AAC AGA ATC TTG C-3′) were used for the first round of PCR amplification, while AmargMSP4Fw (5′-CTG AAG GGG GAG TAA TGG G-3′) and AmargMSP4Rev (5′-GGT AAT AGC TGC CAG AGA TTC C-3′) were used in a nested-PCR reaction, which generated a fragment of 344 bp. The DNA extracted from cattle infected with *A. marginale* (isolate Lushi, GenBank: AJ633048) and sterile water was used as the positive and negative control, respectively. The partial *msp1a* gene containing the tandem repeats of *A. marginale* was further amplified from *msp4*-positive samples by PCR as reported previously [18] with some modifications. The outer primers 1733F (5′-TGT GCT TAT GGC AGA CAT TTC C-3′) and 3134R (5′-TCA CGG TCA AAA CCT TTG CTT ACC-3′) were used in the first reaction as described by Lew et al. [18]. An inner forward primer AM-F2 was designed in highly conserved region of *msp1a* sequences available in GenBank using OligoAnalyzer 3.1 (Integrated DNA Technologies, 2012, Iowa, USA). The inner primers AM-F2 (5′-CGT CTC ACA AGT TTG TAC GCT GTG C-3′, in this study) and 2957R (5′-AAA CCT TGT AGC CCC AAC TTA TCC-3′) were used in the second reaction [18]. The reactions were performed in an automatic thermocycler (Bio-Rad, Hercules, USA) with a final volume of 25 μl containing 2.0 μl template DNA. Thermal cycling comprised 4 min of an initial denaturation at 94 °C, 35 cycles of 94 °C for 30 s, annealing for 30 s (55 °C for 1733F/3134R, 60 °C for MSP43/MSP45, AmargMSP4Fw/AmargMSP4Rev and AM-F2/2957R) and 72 °C for 30–90 s (depending on the target fragments), and a final extension at 72 °C for 10 min. Amplified products were analyzed by 1.0% agarose gel electrophoresis.

### Sequences and statistical analysis

The purified PCR amplicons of *msp4* and *msp1a* genes of *A. marginale* were cloned into pGEM-T Easy vector (Promega, Madison, WI, USA). Two recombinants were selected randomly and sequenced (Genscript, Nanjing, China). Sequence analysis was performed using the BLASTn search and the ClustalW software (DNAStar, Madison, WI, USA). The *A. marginale msp1a* sequences were trimmed and translated to amino acids using CLC Genomics Workbench 7.5.1 (Qiagen, Aarhus, Denmark). The tandem repeats of *A. marginale* Msp1a amino acid sequences were identified and aligned by using the ClustalW software. Statistical analysis was conducted using a Chi-square test in PASW statistics 18.0 (SPSS, Chicago, IL, USA). *P*-values of 0.05 or less were considered statistically significant.

### Nucleotide sequence accession numbers

The sequences obtained in this study were submitted to the GenBank database and provided accession numbers as follows: MF326686 and MF326687 for *msp4* and MF326688–MF326790 for *msp1a*.

## Results

*Anaplasma marginale* DNA was detected in 176 of 557 cattle, with an overall infection rate of 31.6% (Table 1). The infection rates of *A. marginale* varied considerably from 0 to 96.9% in different sampling regions. The infection was detected in 17 of 22 counties, representing all ten provinces included in this study. The infection rate of *A. marginale* in the south-west (67/183, 36.6%) was almost comparable with that in the south-central region (102/300, 34.0%) (*χ* ^2^ = 0.163, *df* = 1, *P* > 0.05), but was significantly higher than in the north-east (7/74, 9.5%) (*χ* ^2^ = 11.621, *df* = 1, *P <* 0.001).

Sequence analysis of *msp4* gene confirmed the infections of *A. marginale* in cattle, and two *msp4* sequence variants with 99.7% similarity were obtained in this study. The *msp4* sequence variant 20-14c (GenBank MF326686) was identical to the *A. marginale* strains Tamaulipas, Kanchanaburi66 and 11-MSP43 (GenBank: EU283844, KU764497 and KX840009) from Mexico, Thailand and China, respectively [19]. The sequence variant 1-15a (GenBank: MF326687) has 99.7–100% identity to strains Nakhonpathom195 and AMSP4-HYD21 (GenBank: KU764498 and KX989532) from Thailand and India, respectively [20].

**Fig. 1:**
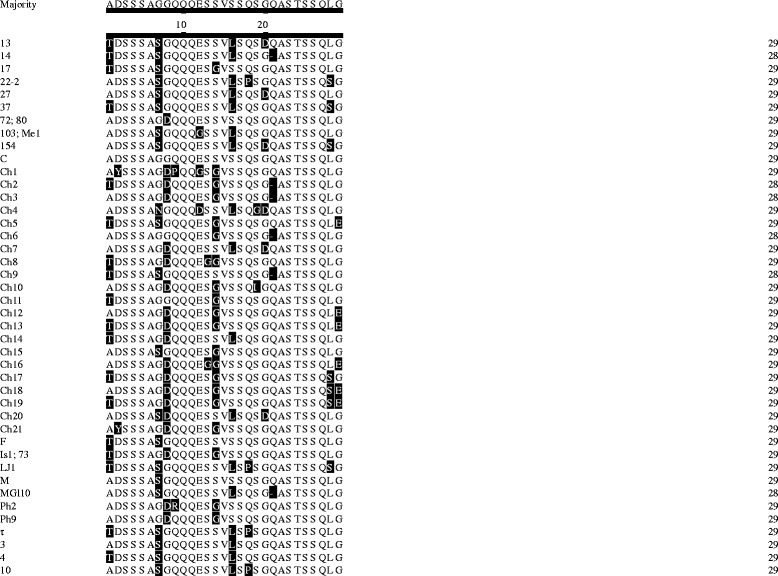
Alignment of Msp1a amino acid repeat sequences of *A. marginale* detected from Chinese cattle. The 42 repeat types were aligned using the ClustalW method in the MegAlign software. The Msp1a tandem repeats (M, F, τ, Ph9, Is1; 73, 13, 27, MGl10, 154, 103; Me1, 14, 72; 80, C, 3, 17, 10, LJ1, 22–2, 37, 4 and Ph2) identified herein have been reported in previous studies, and 21 new Msp1a tandem repeats were named as Ch1–21. The one letter code was used to reveal the different amino acid sequences of Msp1a repeats. The variable amino acids are highlighted on a black background and gaps indicate deletions/insertions

On the basis of the *msp4* PCR results, *A. marginale*-positive samples were subjected for further analysis. One hundred and three *msp1a* sequences (GenBank: MF326688–MF326790) were obtained. Sequence analyses revealed that 97.1% (100/103) of *A. marginale* isolates contained the Msp1a tandem repeats, and 42 different types of Msp1a tandem repeats with 28 to 29 amino acids among Chinese *A. marginale* strains were identified (Fig. 1). Aside from Msp1a tandem repeats (M, F, τ, Ph9, Is1; 73, 13, 27, MGl10, 154, 103; Me1, 14, 72; 80, C, 3, 17, 10, LJ1, 22–2, 37, 4 and Ph2) with known name reported in previous studies [21], 21 new tandem repeats (designated as Ch1–21; Fig. 1) are described for the first time in this study.

The genetic diversity of *A. marginale* strains was analyzed based on the Msp1a tandem repeats structure. A total of 103 *A. marginale* isolates were classified into 61 genotypes with a maximum repeat number of five (Table 2). Interestingly, three isolates (AM5-2a, AM5-2b and AM17-2b; GenBank: MF326718, MF326719 and MF326770) had no amino acid repeats (Table 2). The remaining 100 isolates contained one to five Msp1a tandem repeats. As shown in Table 2, five Msp1a tandem repeats were identified in five *A. marginale* isolates; four repeats in 23 isolates; three repeats in 26 isolates; two repeats in 32 isolates and a single repeat in 14 isolates (Table 2). Most of these Msp1a tandem repeats (Ch1, F, M, Ph9, etc.) were shared between different *A. marginale* isolates and genotypes, while some of them (Ch4, Ch5, Ch7, etc.) were unique and had a low frequency among these isolates (Table 2). In addition, 21 animals positive for *A. marginale* identified in this study were infected by more than one genotype.

**Table 2:** Organization of Msp1a tandem repeats in *A. marginale* strains identified in cattle

Strains	GenBank ID	Structure of Msp1a tandem repeats				
AM1-10a	MF326688	Ch1	M			
AM1-10b	MF326689	Ch1	F	M	M	
AM1-102a	MF326690	Ch1				
AM1-102b	MF326691	Ch1				
AM3-10b	MF326692	τ	M	Ch2		
AM3-21b	MF326693	Ph27	Is1; 73	Is1; 73	Is1; 73	Is1; 73
AM3-21c	MF326694	Ph27	Is1; 73	Is1; 73	Is1; 73	Is1; 73
AM3-27a	MF326695	13	27	27	27	
AM3-27c	MF326696	13	27	27		
AM4-1a	MF326697	Ch1				
AM4-1b	MF326698	Ch1				
AM4-2b	MF326699	Ch3	Ch2	Ch2		
AM4-4a	MF326700	Ch1				
AM4-4c	MF326701	Ch1	M	F	M	
AM4-6a	MF326702	MGl10	154			
AM4-7a	MF326703	2Is1; 73	Is1; 73			
AM4-8a	MF326704	2Is1; 73	Is1; 73			
AM4-9b	MF326705	2Is1; 73	Is1; 73			
AM4-10b	MF326706	2Is1; 73	Is1; 73			
AM4-12a	MF326707	Ch4	Ch5			
AM4-12b	MF326708	Ch4	Ch5			
AM4-15a	MF326709	Ch6	Ch2	Ch2	Ch2	
AM4-15b	MF326710	M	M	103; Me1		
AM4-17b	MF326711	Ph9	Is1; 73			
AM4-18a	MF326712	Ch7	14			
AM4-18b	MF326713	Ch7	14			
AM4-21b	MF326714	72; 80	Ch8	Ch8	Ch8	Ch8
AM4-22b	MF326715	Ch7	14			
AM4-23b	MF326716	27	Is1; 73	Is1; 73		
AM4-24a	MF326717	Ch9	Ch3	Ch3	Ch3	
AM5-2a	MF326718					
AM5-2b	MF326719					
AM5-4a	MF326720	Ch3	Ch2	Ch2		
AM5-4b	MF326721	Ch1	M			
AM5-6a	MF326722	13				
AM5-6b	MF326723	Ch3	Ch2	Ch2	Ch2	
AM5-8a	MF326724	F	M	M		
AM5-8b	MF326725	Ch1				
AM5-9b	MF326726	Ch1	M	F	M	
AM5-11b	MF326727	Ph9	Is1; 73	Ch2		
AM5-11c	MF326728	Ph9	Is1; 73	Is1; 73	Is1; 73	
AM5-13c	MF326729	27	Is1; 73			
AM5-15a	MF326730	27	Is1; 73	Is1; 73		
AM5-15c	MF326731	27	Is1; 73			
AM5-16b	MF326732	F	M	C		
AM5-19a	MF326733	Ch1	M	F	M	
AM5-19b	MF326734	Ch1	M	F	M	
AM5-22a	MF326735	13	14	M		
AM5-22c	MF326736	Ch1	M	F	M	
AM6-7a	MF326737	Ch10	Is1; 73	Is1; 73		
AM6-7b	MF326738	Ch10	Is1; 73	Is1; 73		
AM7-8b	MF326739	F	M	M		
AM8-5c	MF326740	27	Is1; 73	24	Is1; 73	
AM9-3b	MF326741	3				
AM9-3c	MF326742	103; Me1	3	3		
AM9-5a	MF326743	13	17	Ch2		
AM9-14a	MF326744	F	10			
AM9-14b	MF326745	F	10	MG110		
AM9-21a	MF326746	LJ1	22–2	27	14	
AM9-24a	MF326747	M	F			
AM9-24b	MF326748	Ph9	Is1; 73	Is1; 73	Is1; 73	Is1; 73
AM9-26b	MF326749	13				
AM9-26c	MF326750	37	154	27		
AM15-3b	MF326751	Ph9	Is1; 73			
AM15-5a	MF326752	27	Is1; 73	Is1; 73	Is1; 73	
AM15-5b	MF326753	27	Is1; 73	Is1; 73	Is1; 73	
AM15-18a	MF326754	Ch12	Is1; 73			
AM15-18b	MF326755	Ch12	Ch13	Ch13	Is1; 73	
AM15-24a	MF326756	3	Ch2			
AM15-30a	MF326757	M	M	M	M	
AM15-30b	MF326758	13	4			
AM16-1b	MF326759	Ch14	Ch2			
AM16-2b	MF326760	13	14			
AM16-2c	MF326761	13	14			
AM16-5c	MF326762	Ph9				
AM16-9b	MF326763	Ch15	Is1; 73			
AM16-12b	MF326764	Ch10	Is1; 73	Is1; 73		
AM16-14a	MF326765	13	14			
AM16-14b	MF326766	13	14			
AM16-21b	MF326767	13	14			
AM16-25a	MF326768	Ch16	Ch17	Ch17	Is1; 73	Ch2
AM16-25b	MF326769	Ch18	Ch19	Is1; 73	Ch2	
AM17-2b	MF326770					
AM18-3b	MF326771	3	Is1; 73	Is1; 73		
AM18-3c	MF326772	27	Is1; 73	Is1; 73		
AM18-6b	MF326773	Ch20	13			
AM18-8b	MF326774	M	Ch11	Ph9		
AM18-15a	MF326775	13				
AM18-15b	MF326776	4	4	3		
AM18–19a	MF326777	Ch21	Is1; 73	Is1; 73		
AM18–19	MF326778	F	F			
AM18-20b	MF326779	Ch15				
AM18-24b	MF326780	F	M	M	M	
AM19-1b	MF326781	Ph9	Is1; 73	Is1; 73		
AM19-1c	MF326782	Ph9	Is1; 73			
AM19-2b	MF326783	Ph9				
AM19-2c	MF326784	Ph9				
AM19-4b	MF326785	Ph2	Is1; 73	Is1; 73	Is1; 73	
AM19-6a	MF326786	Ph9	Is1; 73	Is1; 73	Is1; 73	
AM19-6b	MF326787	Ph9	Is1; 73	Is1; 73		
AM19-8b	MF326788	Ph9	Is1; 73			
AM19-9c	MF326789	Ph9	Is1; 73	Is1; 73	Is1; 73	
AM19-10a	MF326790	Ph9	Is1; 73	Is1; 73	Is1; 73	

## Discussion

Bovine anaplasmosis caused by *A. marginale* is widely distributed in tropical and subtropical areas throughout the world [22]. In China, *A. marginale* was first isolated from cattle as early as 1987 in Lushi County, Henan Province [11]. Since then, *A. marginale* has been detected in *Hyalomma asiaticum* ticks and cows from five farms in northwestern China [13]. A molecular survey of *Anaplasma* spp. has previously been conducted in domestic ruminants from 12 provinces of China, and *A. marginale* infection in cattle was identified by *gltA* sequencing [14]. In addition, this agent has also been found in cattle from Chongqing, southwestern China [15]. Those reports provided molecular evidence of *A. marginale* by genus-specific PCR and sequencing in domestic ruminants in China. However, information of epidemiology and molecular characterization of Chinese strains is limited. In the present study, a molecular survey of *A. marginale* was conducted by species-specific PCR in cattle, and 31.6% of 557 sampled animals were naturally infected with this organism. Since animals infected by *A. marginale* can develop a persistent infection that may facilitate the maintenance and further spread of infection [23], a high prevalence of *A. marginale* was relatively common in the vertebrate hosts. In this study, a significant difference in infection rates of *A. marginale* was observed between the South and the North area of China, and this may be mainly associated with the tick vectors. The geographical distribution of different tick species in China vary from South to North due to the diverse ecological environments, climate variability and hosts [24], affecting consequently the presence of tick-borne diseases. *Anaplasma marginale* was identified in all ten sampled provinces, indicating that this agent was widely distributed and may pose a serious threat to the cattle industry in China, which should arise extensive attention.

The members in the genus *Anaplasma* have diverse surface-exposed proteins [6]. There are six major surface proteins (MSPs) that have been well characterized in *A. marginale*, and were considered to be involved in the interactions of pathogen with both ticks and hosts [22, 25]. These major surface protein genes may evolve more obviously because of the selective pressure exerted by the host immune system [26]. The genetic variability of *A. marginale* was frequently characterized on the basis of the *msp4* and *msp1a* genes [27]. However, the *msp4* gene is highly conserved and stable among widely divergent strains of *A. marginale* [28]. In this study, the *msp4* sequences of *A. marginale* isolates identified in cattle from different geographical regions shared high sequence identity (99.7 to 100%), and have previously been reported in cattle from other countries [19, 20].

*Anaplasma marginale* geographical strains differing in their biological properties have been genetically characterized, 234 Msp1a tandem repeats were identified and summarized recently by Catanese et al. [21], providing over 350 genotypes based on the structure of Msp1a amino acid repeats [21]. In the present study, a comparison of 103 isolates from different geographical regions permitted identification of 42 Msp1a tandem repeats, 50% of which were identical to those previously published for *A. marginale* strains. The Msp1a tandem repeats were not always clustered together corresponding to the geographical locations; some repeats have been identified in the *A. marginale* isolates from various regions and appeared to be distributed nationwide (Table 2). These findings suggest that there is no significant association between specific Msp1a repeats and geographical regions, and this may be attributed to movement of vectors and vertebrate hosts.

*Anaplasma marginale* geographical strains differ in the copy number and amino acid repeat sequences in Msp1a [29]. In our study, 61 *A. marginale* genotypes were identified based on the variation in the repeated portion of Msp1a, showcasing the broad genetic diversity of *A. marginale* in cattle in China. Previous reports have demonstrated that the Msp1a repeats contain functional domains that are involved in adhesion to tick cells and bovine erythrocytes [30]. They also contain B cell and neutralization epitopes that are critical for immune protection in animals [30], suggesting that Msp1a repeats play an important role in the invasion, transmission and survival of *A. marginale*. Generally, *A. marginale* strains contain at least one Msp1a tandem repeat (maximum number of 10) [6]; however, the repeat sequence was not found in three isolates from Guangdong and Guangxi Province in south-central China.

It has been demonstrated that the animals and ticks naturally infected with one genotype of *A. marginale* preclude infection with additional genotypes, indicating that different genotypes could not coexist in the same animals and ecosystems [31, 32]. This infection exclusion mechanism has also been revealed for *Rickettsia* species [33]. However, *A. marginale* strain superinfection with different Msp1a genotypes has been reported subsequently and proven to be associated with high levels of infection prevalence [34–36]. In the present study, 21 animals positive for *A. marginale* were infected by multiple genotypes. This finding was consistent with the previous report [37], in which described distinct *A. marginale* strains circulated in the same animals and herd. A similar phenomenon was also observed for *A. marginale* subsp. *centrale* [38]. The coexistence of divergent *A. marginale* strains may serve as a potential source of variation.

In summary, our results revealed the prevalence and genetic diversity of *A. marginale* strains using Msp1a tandem repeats in ten provinces. As one of the most important tick-borne diseases, bovine anaplasmosis caused by *A. marginale* should no longer be neglected in endemic areas of China.

## Conclusions

In the present study, 31.6% of 557 cattle from 22 counties of ten provinces were positive for *A. marginale*. The *A. marginale* strains were molecularly characterized based on the structure of Msp1a amino acid repeats. A total of 103 isolates were classified to 61 genotypes, and 42 Msp1a tandem repeats were identified, 21 of which have not previously been described. The present study, for the first time, revealed the genetic diversity of *A. marginale* strains using Msp1a repeat sequences in cattle in China.

## Abbreviations

EDTA: ethylene diamine tetraacetic acid
Msp: major surface protein
UV: ultraviolet
